# Ixazomib in combination with carboplatin in pretreated women with advanced triple-negative breast cancer, a phase I/II trial of the AGMT (AGMT MBC-10 trial)

**DOI:** 10.1186/s12885-018-4979-0

**Published:** 2018-11-06

**Authors:** Gabriel Rinnerthaler, Simon Peter Gampenrieder, Andreas Petzer, Sonja Burgstaller, David Fuchs, Dieter Rossmann, Marija Balic, Daniel Egle, Holger Rumpold, Christian F. Singer, Rupert Bartsch, Edgar Petru, Thomas Melchardt, Hanno Ulmer, Brigitte Mlineritsch, Richard Greil

**Affiliations:** 1 IIIrd Medical Department with Hematology and Medical Oncology, Hemostaseology, Rheumatology and Infectious Diseases, Oncologic Center, Paracelsus Medical University Salzburg, Müllner Hauptstrasse 48, 5020 Salzburg, Austria; 2 Salzburg Cancer Research Institute with Laboratory of Immunological and Molecular Cancer Research and Center for Clinical Cancer and Immunology Trials, Salzburg, Austria; 3 Cancer Cluster Salzburg, Salzburg, Austria; 4 Internal Department I for Medical Oncology and Hematology, Ordensklinikum Linz Barmherzige Schwestern, Linz, Austria; 5 IVth Department of Internal Medicine with Hematology and Medical Oncolocy, Klinikum Wels-Grieskirchen, Wels, Austria; 6 Department of Internal Medicine 3 - Hematology and Oncology, Kepler University Hospital, Linz, Austria; 7 2nd Medical Department, County Hospital Steyr, Steyr, Austria; 8 Division of Oncology, Department of Internal Medicine, Medical University Graz, Graz, Austria; 9 Department of Obstetrics and Gynaecology, Innsbruck Medical University, Innsbruck, Austria; 10 Department of Oncology, Hematology and Gastroenterology, Academic Teaching Hospital Feldkirch, Feldkirch, Austria; 11 Department of Obstetrics and Gynecology, Cancer Comprehensive Center, Medical University of Vienna, Vienna, Austria; 12 Department of Internal Medicine 1, Division of Oncology, Cancer Comprehensive Center, Medical University of Vienna, Vienna, Austria; 13 Department of Obstetrics and Gynaecology, Clinical Department of Gynecology, Medical University Graz, Graz, Austria; 14 Department of Medical Statistics and Informatics, Medical University Innsbruck, Innsbruck, Austria

**Keywords:** Triple-negative, Breast cancer, Ixazomib, Carboplatin, Platinum, Proteasome inhibitor

## Abstract

**Background:**

Triple-negative breast cancer (TNBC) comprises a heterogeneous group of diseases which are generally associated with poor prognosis. Up to now, no targeted treatment beyond anti-VEGF therapy has been approved for TNBC and cytotoxic agents remain the mainstay of treatment. Ixazomib is a selective and reversible inhibitor of the proteasome, which has been mainly investigated in the treatment of multiple myeloma. In a preclinical study TNBC cells were treated with the first-generation proteasome inhibitor bortezomib in combination with cisplatin and synergistic efficacy was demonstrated. Clinical data are available for carboplatin plus bortezomib in metastatic ovarian and lung cancers showing remarkable antitumor activity and good tolerability (Mol Cancer 11:26 2012, J Thorac Oncol 4:87–92 2009, J Thorac Oncol 7:1032–1040, 2012). Based on this evidence, the phase I/II MBC-10 trial will evaluate the toxicity profile and efficacy of the second-generation proteasome inhibitor ixazomib in combination with carboplatin in patients with advanced TNBC.

**Methods:**

Patients with metastatic TNBC pretreated with at least one prior line of chemotherapy for advanced disease with a confirmed disease progression and measurable disease according to RECIST criteria 1.1 are eligible for this study. Patients will receive ixazomib in combination with carboplatin on days 1, 8, and 15 in a 28-day cycle. The phase I part of this study utilizes an alternate dose escalation accelerated titration design. After establishing the maximum tolerated dose (MTD), the efficacy and safety of the combination will be further evaluated (phase II, including 41 evaluable patients). All patients will continue on study drugs until disease progression, unacceptable toxicity or discontinuation for any other reason. Primary endpoint of the phase II is overall response rate, secondary endpoints include progression-free survival, safety, and quality of life. This trial is open for patient enrollment since November 2016 in six Austrian cancer centers. Accrual is planned to be completed within 2 years.

**Discussion:**

Based on preclinical and clinical findings an ixazomib and carboplatin combination is thought to be effective in metastatic TNBC patients. The MBC-10 trial is accompanied by a broad biomarker program investigating predictive biomarkers for treatment response and potential resistance mechanisms to the investigational drug combination.

**Trial registration:**

EudraCT Number: 2016–001421-13 received on March 31, 2016, ClinicalTrials.gov Identifier: NCT02993094 first posted on December 15, 2016. This trial was registered prospectively.

## Background

Metastatic breast cancer is still an incurable disease and significantly contributes to worldwide cancer mortality [1]. Prognosis clearly depends on the molecular subtype determined either by immunohistochemistry or molecular assays as well as on proliferation rate as determined by the Ki-67 index. By the introduction of novel endocrine agents, targeted agents and immunotherapy, a substantial progress has been made for both hormone-receptor-positive as well as Her2/neu positive metastatic breast cancers [2], although resistance might still exist either primarily or develop during or after therapy.

Triple-negative breast cancer (TNBC) comprises a heterogeneous group of diseases, defined by the absence of an estrogen receptor, progesterone receptor and HER2 expression. TNBC accounts for approximately 15% of breast cancer cases and is now the subtype with the worst prognosis and novel concepts are urgently needed. No targeted treatment beyond anti-VEGF therapy is approved for TNBC so far and cytotoxic agents are the mainstay for the treatment of the advanced disease. Up to 20% of patients with TNBC harbor a germline BRCA mutation [3]. Recently, the results of the phase III trial OlympiAD comparing olaparib, an oral poly(ADP-ribose) polymerase (PARB) inhibitor with standard chemotherapy in pretreated metastatic breast cancer patients harboring a germline BRCA mutation have been published showing favorable efficacy and safety results for the PARP inhibitor [4]. Therefore, an approved target treatment option for a subset of TNBC patients can be awaited within the next year. Due to a generally aggressive course of disease there is an urgent need for the investigation of novel drugs and drug combinations.

### Rationale for the use of ixazomib in breast cancer

Ixazomib is a selective, potent, and reversible inhibitor of the proteasome, which has been investigated for the treatment of multiple myeloma [5]. In solid tumors, the cytotoxic effect of proteasome inhibitors is thought to be mediated through different mechanisms: (1) Inhibition of the Fanconi Anemia (FA) and BRCA1 DNA repair mechanism [6]. The FA signaling cascade coordinates a complex mechanism consisting of three classical DNA repair systems (homologous recombination, nucleotide excision repair, mutagenic translesion synthesis) in response to DNA damage [7]: Five FA proteins (A, C, E, F, and G) regulate the activation of FANCD2 by monoubiquitination and nuclear foci formation. Activated FANCD2 [8] as well as BRCA1 regulate the DNA repair by homologous recombination [9] and are therefore critical for the function of the Fanconi anemia pathway [7]. In addition, FANCD2 interacts with the MRE11-NBS1-RAD50 complex in the repair of DNA crosslinks [7]. Proteasome inhibitors like bortezomib have been shown to inhibit monoubiquitination and/or nuclear foci formation of FANCD2 thereby causing cellular hypersensitivity to ionized radiation and DNA cross-linking agents like platinum salts [10, 11]. (2) Inhibition of p53 degradation. Inhibition of the ubiquitin-proteasome mediated degradation of p53 [7, 12, 13] as well as of the natural cyclin dependent-kinase inhibitors p21 and p27, induces G1 arrest. (3) Induction of apoptosis by inhibition of the canonical and non-canonical NF-kappa B signaling cascade [12–14].

### Rationale for the use of carboplatin in breast cancer

Carboplatin is a platinum based compound that covalently binds with DNA to form intrastrand crosslinks and changes DNA conformation, therefore affecting DNA replication. Compared to cisplatin, carboplatin has a favorable toxicity profile, particularly with less nephrotoxic and neurotoxic side effects but at the price of an increased hematotoxicity [15].

In metastatic breast cancer single agent carboplatin was investigated in several phase II trials including patients irrespective of breast cancer subtype. Response rates were in the range of 20–35% in treatment-naive patients [16–18]. In patients pretreated with chemotherapy response rates of single-agent carboplatin were modest with ORR of lower than 10% in two small phase II trials including 13 and 14 patients, respectively [18, 19]. In a large randomized phase III trial, carboplatin was compared with docetaxel as first line treatment in patients with TNBC or BRCA1/2 mutation-associated breast cancer [20]. Overall response rate, the primary endpoint of this trial, was 31% (59/188) in the carboplatin group and 36% (67/188) in the taxane group. In a preplanned subgroup analysis, overall response rate in the carboplatin arm was 68% (17/25) in BRCA1/2 germline mutated patients compared to 28% (36/128) in patients without germline BRCA1/2 mutation. Combination regimens with carboplatin plus taxanes, anthracyclines, nucleoside analogues, vinca alkaloids, or targeting therapies like poly-adenosine diphosphate-ribose polymerase (PARP)-inhibitors were more promising [21–23]. Based on several phase II trials with response rates in the range of 17–39% [24–28], the combination of carboplatin plus gemcitabine is a clinically established treatment option for pretreated patients with advanced triple-negative breast cancer.

### Rationale for a combination of ixazomib and carboplatin

In a preclinical study the triple-negative breast cancer cell line 4 T1 was treated with bortezomib alone and in combination with cisplatin. The combination of both drugs led to a higher inhibition of cell growth than each drug alone thus suggesting a synergistic effect [29].

TNBC is a rather heterogeneous group of tumors when analyzed with molecular and immunological tools [30–32]. When analyzed at the molecular level, it is mostly categorized within the so called basal-like subtype [30]. The Total Cancer Genome Atlas (TCGA) network uncovered the heterogeneity of genetic alterations in breast tumors and showed a remarkable similarity of basal-like tumors with serous ovarian cancers [33]. Common features between serous ovarian and basal-like tumors are BRCA1 inactivation, RB1 loss and cyclin E1 amplification, high expression of AKT3, high expression and amplification of MYC, and a high frequency of TP53 mutations. Compared to luminal breast cancers, TNBC also have a reduced DNA repair capability due to a reduced expression of DNA repair genes including nucleotide excision repair, Fanconi Anemia pathways and CHK1 [34]. These particular molecular features might be exploited in novel targeted approaches and simultaneously indicate potential overlapping strategies in the treatment of breast and ovarian cancers, with platinum compounds representing the major backbone of treatment for the latter.

Bortezomib in combination with carboplatin was tested in 15 patients with recurrent ovarian cancer in a phase I trial, showing a remarkable antitumor activity with an overall response rate of 47% (2 CR and 5 PR) [35]. One patient suffered from platinum-resistant disease but achieved a complete remission with this combination. Interestingly, bortezomib decreased the serum level of NF-kB, which is known to be increased by carboplatin, in seven of eight patients with increased NF-kB activity after carboplatin infusion. Re-exposure to carboplatin couldn’t reactivate NF-kB during the following cycle, indicating a lasting effect of bortezomib in this regard.

The combination of bortezomib and carboplatin was also tested in 22 patients with platinum- and taxane-resistant ovarian cancer [36]. In these heavily pretreated patients no objective response was seen, but 44% (8 of 18 evaluable patients) had a stable disease. Based on these data, this drug combination could show efficacy in triple-negative breast cancers, known to be biologically similar to ovarian cancer.

The bortezomib and carboplatin regimen was combined with gemcitabine in a triple drug first line regimen in a phase II trial in patients with advanced non-small cell lung cancer (*n* = 114) [37] and in the same indication in combination with bevacizumab within a phase I/II trial (*n* = 16) [38]. The leading grade 3 and 4 toxicities occurring in more than 15% of patients were thrombocytopenia (63%) and neutropenia (52%) in combination with gemcitabine, and thrombocytopenia (58%), lymphocytopenia (25%), diarrhea (25%), nausea (19%), and vomiting (19%) in combination with bevacizumab. Response rates were 23% in the gemcitabine containing trial and 44% in the bevacizumab containing trial [37, 38].

## Methods

### Study design

**Fig. 1:**
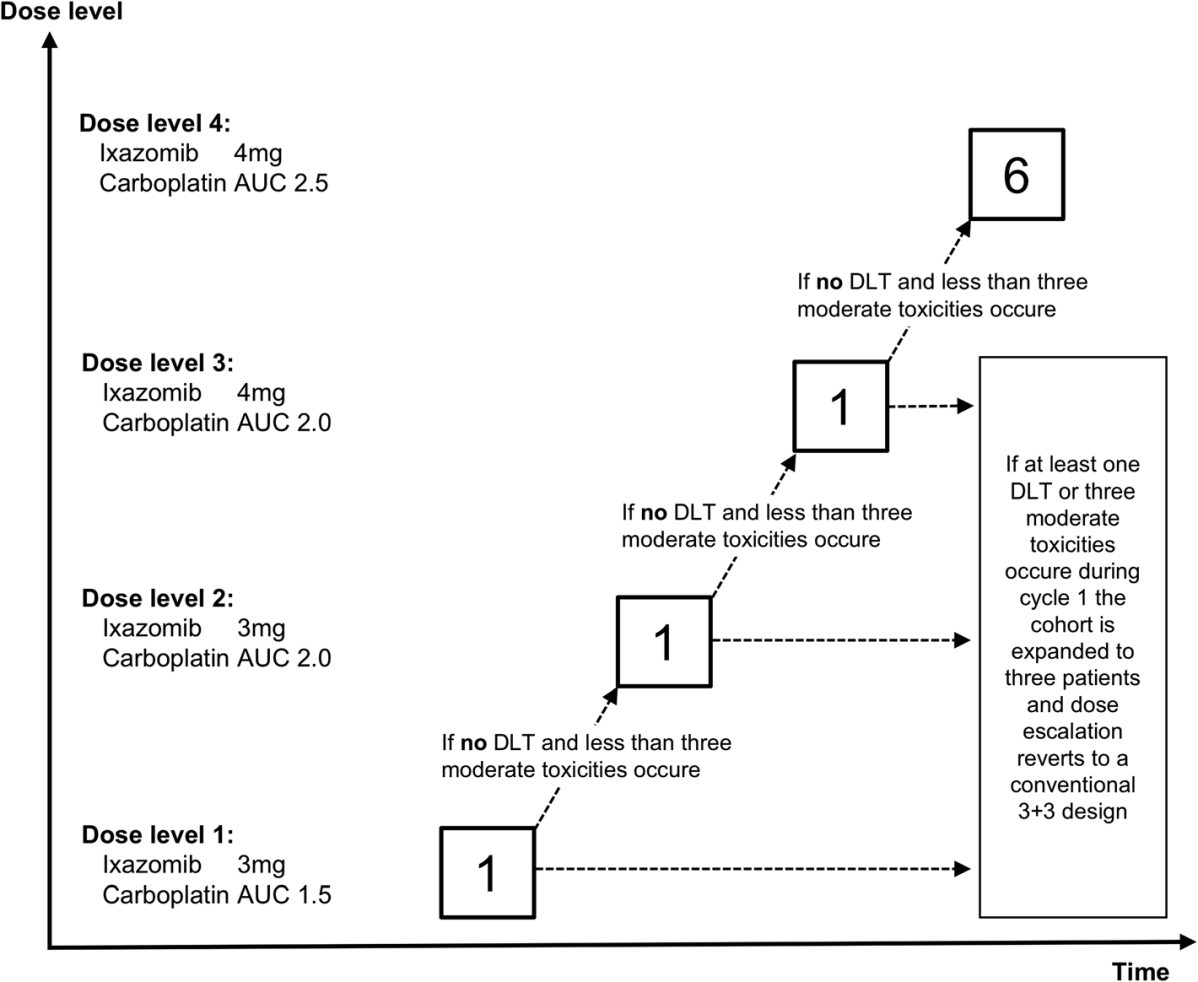
Accelerated titration design of the phase I part of the trial. Illustration of the accelerated titration design of the phase I part of the trial. Each box outlined in bold represents a cohort containing the indicated number of patients treated at a given dose level. Ixazomib will be administrated orally on days 1, 8, and 15 and carboplatin intravenously on days 1, 8, and 15 of a 4-week cycle. Drug dosages per dose level are given on the left side of the graph. DLT = dose-limiting toxicity; AUC = area under the curve

The phase I part of this study uses an alternate dose escalation accelerated titration design [39] as outlined in Fig. 1 and Table 1. In the accelerated dose-escalation phase a single-patient cohort per dose level will be enrolled, until one dose limiting toxicity (DLT) or 3 moderate toxicities, as outlined below, are observed during cycle 1, or until dose level 4 is reached. At this dose level the cohort is expanded to three patients and dose escalation reverts to a conventional 3 + 3 escalation design [40]. Dose limiting toxicities (DLTs) are defined as drug related adverse events preventing the administration of the drug combination. Such toxicities are defined as grade 3 or 4 non-hematologic toxicities (excluding alopecia, nausea, emesis, diarrhea), grade 3 or greater nausea and/or emesis despite the use of optimal anti-emetic prophylaxis, grade 3 or greater diarrhea that occurs despite maximal supportive therapy, grade 2 peripheral neuropathy with pain or polyneuropathy greater or equal grade 3, neutropenia grade 4 for more than 7 days, febrile neutropenia grade 3, thrombocytopenia grade 4, or thrombocytopenia grade 3 with bleeding. Moderate toxicities are defined as any grade 2 non-hematologic toxicity excluding alopecia, or any grade 3 hematologic toxicity.

**Table 1:** Drug dosages per dose level

Dose level	Ixazomib orally on days 1, 8, 15 every 4 weeks	Carboplatin i.v. on days 1,8 and 15 every 4 weeks
1	3 mg	AUC 1.5
2	3 mg	AUC 2
3	4 mg	AUC 2
4	4 mg	AUC 2.5

AUC = area under the curve

The maximum-administered dose (MAD) is defined as the dose at which DLT or 3 moderate toxicities occur in at least two of six patients treated at that dose level. The dose-level just below the MAD is considered the maximum-tolerated dose (MTD), providing that DLT or 3 moderate toxicities are observed in fewer than two of six treated patients (or fewer than one third if more than six patients will be treated) at that dose level. Determination of MAD and MTD is based on DLT or 3 moderate toxicities observed during the first treatment cycle.

Beginning with the 2nd cycle an intra-patient dose escalation is allowed. This is separate from the alternate dose escalation design of the phase I part. If the nadir ANC was ≥500/mL, the nadir platelet count ≥50,000/mL, and non-hematologic toxicities were grade ≤ 1 in the previous cycle, the current dose level can be escalated by one dose level by each cycle. After completion of phase I, all patients, that are treated with a dose below the determined MTD, can be dose escalated at the discretion of the investigator [39, 41].

After establishing MTD in phase I, accrual continues in phase II to evaluate the efficacy and safety of the combination therapy. A total of 41 evaluable patients will be included (patients enrolled in the phase I part within the conventional dose escalation phase at the dose level considered as the MTD may be included).

All subjects will continue on study drugs until disease progression, unacceptable toxicity or treatment discontinuation for any other reason, respectively.

### Study population

Overall, a maximum of 53 patients will be included. Of them, a maximum of 24 will be treated in the dose escalation phase and 41 patients in the phase II part of the study.

### Patients

Patients with metastatic histologically confirmed triple-negative adenocarcinoma of the breast will be assessed for eligibility. Detailed inclusion and exclusion criteria are listed in Table 2. Patients with locally advanced disease are eligible provided they cannot be offered a potentially curative loco-regional treatment option. TNBC is defined by the absence of staining for estrogen receptor (IHC < 1%), progesterone receptor (IHC < 1%) and HER2/neu (IHC 1+ or ISH ratio of < 2.0 between HER2 gene copy number and centromere of chromosome 17 or a copy number of 4 or less [42]). A signed informed consent, with the understanding that consent may be withdrawn at any time without prejudice to future medical care, have to be provided prior to any study-specific procedure. All patients must be female and aged 18 or older with a performance status of ECOG 0–2 and a life expectancy of at least 12 weeks. A disease progression with at least one measurable lesion according to RECIST 1.1 criteria must be documented. Patients must be pretreated with at least one prior line of chemotherapy for advanced disease or a progression must have occurred within 12 months of completion of adjuvant chemotherapy. Women of childbearing potential must have a negative pregnancy test at screening and agree to practice 2 effective methods of contraception, at the same time. An adequate left ventricular ejection fraction at baseline, defined as LVEF ≥50% by either echocardiogram or MUGA and adequate hematological, liver and renal function (absolute neutrophil count ≥1.5 × 10^9^/L, hemoglobin ≥8 g/dL, platelets ≥100 × 10^9^/L, albumin ≥2.5 g/dL, serum bilirubin ≤2 mg/dL, aspartate transaminase and alanine transaminase ≤3 x ULN without liver metastases and ≤ 5 x ULN with documented liver metastases, and serum creatinine ≤1.5 mg/dL or calculated creatinine clearance ≥50 ml/min) is required. If present, a peripheral neuropathy must be less or equal to grade 1 or grade 2 without pain.

**Table 2:** Detailed Inclusion and Exclusion criteria

Inclusion criteria	Exclusion criteria
Each patient must meet all of the following inclusion criteria to be enrolled in the study: • Metastatic or locally advanced (without curative loco-regional treatment options with curative intention) adenocarcinoma of the breast, histologically confirmed • Triple-negative subtype defined as the absence of staining for estrogen receptor (IHC < 1%), progesterone receptor (IHC < 1%) and HER2/neu (IHC 0–1+, or 2+ if FISH-test is negative, or ISH ratio of < 2.0 between Her2 gene copy number and centromere of chromosome 17 or a copy number of 4 or less) • Signed informed consent prior to any study-specific procedure, with the understanding that consent may be withdrawn at any time without prejudice to future medical care • Female patients, age ≥ 18 years • Women of childbearing potential must have a negative pregnancy test at screening and agree to practice 2 effective methods of contraception, at the same time, from the time of signing the informed consent form through 90 days after the last dose of study drug OR agree to practice true abstinence when this is in line with the preferred and usual lifestyle of the subject (Periodic abstinence [eg, calendar, ovulation, symptothermal, post-ovulation methods] and withdrawal are not acceptable methods of contraception. • At least one prior line of chemotherapy for metastatic or locally advanced disease or disease progression within 12 months of completion of adjuvant chemotherapy • Documented disease progression • At least one measurable lesion according to RECIST 1.1 criteria • Life expectancy of at least 12 weeks • Performance status ECOG 0–2 • Adequate left ventricular ejection fraction at baseline, defined as LVEF ≥50% by either echocardiogram or MUGA • Peripheral neuropathy NCI CTCAE grade ≤ 1 or grade 2 if no pain on clinical examination • Adequate hematological, liver and renal function: Hematologic: ANC (absolute neutrophil count) ≥ 1.5 × 109/L Hemoglobin ≥8 g/dL Platelets ≥100 × 109/L Liver Function: Albumin ≥2.5 g/dL Serum bilirubin ≤2 mg/dL AST and ALT ≤3 x ULN without liver metastases and ≤ 5 x ULN with documented liver metastases Renal Function: Serum creatinin ≤1.5 mg/dL or calculated creatinin clearance ≥50	Patients meeting any of the following exclusion criteria are not to be enrolled in the study. • Pregnant or lactating women • Serious medical or psychiatric disorders that would interfere with the patient’s safety or informed consent • Clinically significant cardiovascular disease, requiring medication during the study and which might interfere with regularity of the study treatment, or not controlled by medication. • Radiation of the target lesion within the last 4 weeks prior to randomization • Prior radiation to ≥30% of bone marrow • Active bacterial, viral or fungal infection • Known HIV infection • Patients with clinically apparent brain metastases or evidence of a spinal cord compression • Major surgery within 14 days before enrollment • Systemic treatment, within 14 days before the first dose of ixazomib, with strong CYP3A inducers (rifampin, rifapentine, rifabutin, carbamazepine, phenytoin, phenobarbital), or use of *Ginkgo biloba* or St. John’s wort. • Participation in other clinical trials, including those with other investigational agents not included in this trial, within 30 days of the start of this trial and throughout the duration of this trial • Patients that have previously been treated with ixazomib, or participated in a study with ixazomib whether treated with ixazomib or not • History of other malignancy; patients who have been disease-free for 5 years or patients with a history of completely resected non-melanoma skin cancer or successfully treated in situ carcinoma are eligible • Prior treatment with a platinum derivative (except in (neo-)adjuvant setting if breast cancer recurrence did not occur within 12 months after (neo-)adjuvant chemotherapy completion) and/or with a proteasome inhibitor • Known hypersensitivity to the study drugs

Patients with at least one of the following conditions or pretreatments are excluded from trial participation: serious medical or psychiatric disorders interfering with patient’s safety or informed consent, lactating patients, radiation of the target lesion within the last 4 weeks before enrollment, prior radiation to ≥30% of bone marrow, active bacterial, viral or fungal infections, known HIV infection, major surgery within 14 days before enrollment, clinically apparent brain metastases or evidence of a spinal cord compression, systemic treatment with strong CYP3A inducers or use of *Ginkgo biloba* or St. John’s wort within 14 days before the first dose of ixazomib, previous treatment with a proteasome inhibitor or with a platinum derivative unless applied in the (neo-)adjuvant setting if breast cancer recurred later than 12 months after (neo-)adjuvant chemotherapy completion, participation in other clinical trials, application of any investigational agents within 30 days of the start and throughout the duration of this trial, history of another malignancy unless disease-free for ≥5 years or with a history of completely resected non-melanoma skin cancer or a successfully treated in situ carcinoma.

### Translational research program

In a translational research project accompanying the MBC-10 clinical study, predictive markers for treatment response to the investigated drug combination of ixazomib plus carboplatin will be evaluated. Further, analyses of resistance mechanisms are planned. Therefore, somatic as well as germline mutations in BRCA1 and BRCA2, BRCA1/2 methylation status, circulating tumor DNA (ctDNA), gene expression profiles and microRNA expressions will be analyzed from tumor tissue and blood samples, respectively. For further analyses, patients´ blood samples (ETDA and serum) will be collected before treatment start, on day 8 of the first four-weekly treatment cycle as well as on day 1 of every subsequent treatment cycle and at the end of study.

### Determination of sample size

In phase I, an accelerated dose-escalation scheme will be used as outlined above. Single patient cohorts will be enrolled until one dose limiting toxicity (DLT) or 3 moderate toxicities are observed until a certain dose level is reached. Toxicities will be classified according to Common Terminology Criteria for Adverse Events (CTCAE) Version 4.03 [43]. At this dose level the cohort is expanded to three patients and dose escalation reverts to a conventional 3 + 3 escalation design. The 3 + 3 design is rule-based with a statistical power of greater than 87% to detect at least one out of three patients with a DLT or 3 moderate toxicities when the probability for a DLT is 50%.

In the phase II study a single-arm two-stage Simon’s design will be used to allow for early termination if unsatisfactory efficacy results are observed. Sample size estimation is based on overall response rate (ORR).

In a large randomized phase III trial carboplatin was compared with docetaxel as first line treatment in patients with triple-negative breast cancer [20]. In the carboplatin group overall response rate as the primary endpoint of this trial was 31.4% (59/188). In our trial, the investigated drug combination will be mainly tested as second or third-line treatment, and response rates are well known to decrease with increasing lines of therapies [44]. Based on these findings, we would expect an ORR of 20 to 25% for a carboplatin monotherapy, and consider a 35–40% response rate a clinically meaningful improvement [45].

In the phase II part of the study a single-arm two-stage Simon’s Design will be used to allow for early termination if unsatisfactory efficacy results are observed. Sample size estimation is based on overall response rate (ORR).

In a two-stage Simon’s design testing a null hypothesis proportion of 0.2 versus an alternative hypothesis proportion of 0.35 in ORR with α = 0.1 and 1-β = 0.80 a total of 41 patients will be required. In the first stage, 22 patients will be accrued and treated. The study will be stopped if there are fewer than five patients with either CR or PR. If there are at least five responses an additional 19 patients will be enrolled and treated until a total of 41 patients. The regimen is concluded to be effective if 12 or more responses out of 41 are observed at the end of the trial.

Assuming a 10% drop-out rate, 46 patients have to be enrolled for phase 2. The maximum number of patients through phases I and II combined will be 53.

### Efficacy analysis

ORR will be assessed according to RECIST v1.1 [46] and comprise partial response (PR) or better occurring during any time of the treatment period. ORR will be given as proportion together with its 95% confidence intervals (CI). PFS and OS will follow standard definitions and will be estimated using the Kaplan-Meier method. Median PFS and OS will be given together with their 95% CI.

Quality of Life (QoL) will be assessed using the validated EORTC QLQ-C30 and EORTC QLQ-BR23 questionnaires [47, 48]. QoL scales will be derived from single item answers and summarized using appropriate descriptive statistics.

Efficacy will be analysed on the basis of intention to treat (ITT) and thus on all patients enrolled in the study. In parallel, a modified ITT approach will be used in which all patients who received at least one dose of study drugs and who were clinically evaluable [with or without a CT-scan]) will be included. In a separate analysis the per-protocol populations will be analysed which are defined as all patients who received at least one dose of study treatment without major protocol deviations. Major protocol deviations will be determined and documented prior to database lock. The analysis of the modified ITT population will be considered as the primary analysis.

### Safety analysis

Safety evaluations will be based on the incidence, type, severity and consequences (e.g. study discontinuation) of an adverse event (AE) as well as on clinically significant changes in patient’s physical examination, vital signs, and clinical laboratory results. Statistical analysis includes descriptive tabulation using measures which are absolute and relative frequencies for categorical data and means, standard deviations, medians and interquartile ranges for continuous data. Further analyses in the phase I study include a description of toxicities regarding frequency, grade, cycle and dose according to Common Terminology Criteria for Adverse Events (CTCAE) Version 4.03 [43].

All safety analyses will be performed on the safety population. The safety population will include all patients who received at least one dose of study drug.

## Discussion

Inhibition of DNA repair mechanisms by proteasome inhibitors could sensitize triple-negative breast tumors to DNA damaging agents like platinum salts. Within the AGMT MBC-10 trial we utilize an innovative straight forward trial concept supporting an expedite early development for the drug combination of ixazomib and carboplatin for the treatment of TNBC. For both, carboplatin and ixazomib, the single agent maximum tolerable dose (MTD) is already known. In order to reduce the number of patients treated at sub-effective doses we use an accelerated titration design in which a single patient will be enrolled per dose level [39]. If toxicities during cycle 1 are outside the predefined acceptable range, or dose level 4 have been reached, the cohort is expanded to three patients and dose escalation reverts to a conventional 3 + 3 design. During the phase I part of the trial, an intra-patient dose escalation will provide the individual patient with a higher chance to reach effective drug dosages. Such escalation is allowed beginning with cycle 2 if toxicities in the previous cycles were within a predefined range [39, 41]. After determining the MTD, patient enrollment will seamless continue into the phase II part of the trial without any delay due to regulatory issues. This trial is accompanied by a translational research program, aimed to identify a subgroup of patients benefiting most within this heterogeneous disease and to decipher resistance mechanisms to this novel drug combination.

## Conclusion

Based on preclinical and clinical findings an ixazomib and carboplatin combination is thought to be effective in advanced triple-negative breast cancer. The objective of the phase I part of the AGMT MBC-10 trial is to determine the maximum tolerable dose of ixazomib in combination with carboplatin in this patient population. In the phase II part of the trial efficacy and tolerability of this treatment combination will be determined. The AGMT MBC-10 trial is accompanied by a broad biomarker program investigating predictive biomarkers for treatment response and potential resistance mechanisms to the investigational drug combination.

## Abbreviations

AE: Adverse event
AGMT: Arbeitsgemeinschaft Medikamentöse Tumortherapie
ALT: Alanine aminotransferase
ANC: Absolute neutrophil count
AST: Aspartate aminotransferase
AUC: area under the curve
BRCA: Breast Cancer Gene
CR: Complete response
CT: Computer tomography
DLT: Dose limiting toxicity
DNA: Deoxyribonucleic acid
ECOG: Eastern cooperative oncology group
EDTA: Ethylenediaminetetraacetic acid
FA: Fanconi Anemia
HIV: Human immunodeficiency virus
ICH: International conference on harmonization
ITT: Intention to treat
MAD: Maximum-administered Dose
MBC: Metastatic breast cancer
MTD: Maximum tolerated dose
MUGA: Multiple-gated acquisition
NCI-CTCAE: National Cancer Institute – Common Terminology Criteria for Adverse Events
ORR: Overall response rate
PR: Partial remission
QoL: Quality of Life
RECIST: Response evaluation criteria in solid tumors
TCGA: Total Cancer Genome Atlas
TNBC: Triple-negative breast cancer
ULN: Upper limit of normal
VEGF: Vascular endothelial growth factor
